# Heterologous expression and processing of the flavescence dorée phytoplasma variable membrane protein VmpA in *Spiroplasma citri*

**DOI:** 10.1186/s12866-015-0417-5

**Published:** 2015-04-02

**Authors:** Joël Renaudin, Laure Béven, Brigitte Batailler, Sybille Duret, Delphine Desqué, Nathalie Arricau-Bouvery, Sylvie Malembic-Maher, Xavier Foissac

**Affiliations:** INRA, UMR 1332 Biologie du Fruit et Pathologie, Villenave d’Ornon, France; Université de Bordeaux, UMR 1332 Biologie du Fruit et Pathologie, Villenave d’Ornon, France; Université de Bordeaux, UMS3420, Bordeaux Imaging Center, Bordeaux, France; CNRS, Bordeaux Imaging Center, UMS 3420 Bordeaux, France; INSERM, Bordeaux Imaging Center, US 004 Bordeaux, France

**Keywords:** Phytoplasma, Flavescence dorée, Variable membrane protein, *Spiroplasma citri*, Surface expression, Protein secretion, Phytoplasma effector

## Abstract

**Background:**

Flavescence dorée (FD) of grapevine is a phloem bacterial disease that threatens European vineyards. The disease is associated with a non-cultivable mollicute, a phytoplasma that is transmitted by the grapevine leafhopper *Scaphoideus titanus* in a persistent, propagative manner. The specificity of insect transmission is presumably mediated through interactions between the host tissues and phytoplasma surface proteins comprising the so-called variable membrane proteins (Vmps). Plant spiroplasmas and phytoplasmas share the same ecological niches, the phloem sieve elements of host plants and the hemocoel of insect vectors. Unlike phytoplasmas, however, spiroplasmas, and *Spiroplasma citri* in particular, can be grown in cell-free media and genetically engineered. As a new approach for studying phytoplasmas-insect cell interactions, we sought to mimic phytoplasmas through the construction of recombinant spiroplasmas exhibiting FD phytoplasma Vmps at the cell surface.

**Results:**

Here, we report the expression of the FD phytoplasma VmpA in *S. citri*. Transformation of *S. citri* with plasmid vectors in which the *vmpA* coding sequence was under the control of the *S. citri tuf* gene promoter resulted in higher accumulation of VmpA than with the native promoter. Expression of VmpA at the spiroplasma surface was achieved by fusing the *vmpA* coding sequence to the signal peptide sequence of the *S. citri* adhesin ScARP3d, as revealed by direct colony immunoblotting and immunogold labelling electron microscopy. Anchoring of VmpA to the spiroplasma membrane was further demonstrated by Triton X-114 protein partitioning and Western immunoblotting. Using the same strategy, the secretion of free, functionally active β-lactamase (used as a model protein) into the culture medium by recombinant spiroplasmas was achieved.

**Conclusions:**

Construction of recombinant spiroplasmas harbouring the FD phytoplasma variable membrane protein VmpA at their surface was achieved, which provides a new biological approach for studying interactions of phytoplasma surface proteins with host cells. Likewise, the secretion of functional β-lactamase by recombinant spiroplasmas established the considerable promise of the *S. citri* expression system for delivering phytoplasma effector proteins into host cells.

**Electronic supplementary material:**

The online version of this article (doi:10.1186/s12866-015-0417-5) contains supplementary material, which is available to authorized users.

## Background

Flavescence dorée (FD) is a quarantine, phloem bacterial disease that threatens European vineyards, especially in France and Italy where wine growing has strong economic and cultural impacts [1,2]. A cell wall-less bacterium belonging to the genus ‘*Candidatus* Phytoplasma’ of the class *Mollicutes* is associated with the disease and is considered to be the causal agent. Phytoplasmas represent an important group of plant pathogenic bacteria, as they cause severe diseases in a wide variety of crops worldwide [3,4]. Phytoplasmas inhabit the phloem sieve tubes and are transmitted from plant to plant by hemipteran, phloem sap-feeding insects [5], which are, therefore, responsible for the spread of diseases.

Studying the molecular components that govern interactions of phytoplasmas with their host plants and insects is severely limited because phytoplasmas cannot be cultured in cell-free media and, thus, cannot be genetically engineered. Nevertheless major breakthroughs have been accomplished through the acquisition of complete genome sequences [6-10] and the functional characterization of phytoplasma secreted proteins, including membrane-associated surface proteins and effector proteins involved in insect transmission and plant pathogenicity [11-13]. For example, the immunodominant protein Amp of ‘*Candidatus* phytoplasma asteris’ strain OY has been shown to specifically interact with actin microfilaments of leafhopper vectors, but not with those of non-vector insects [14]. Similarly, in ‘*Ca*. P. asteris’ strain CYP, Amp interacts with actin and ATP synthase of leafhopper vectors, suggesting that this protein plays a critical role in insect transmission specificity [15], whereas in ‘*Ca*. Phytoplasma mali’, the immunodominant membrane protein Imp specifically binds to plant actin, which supports the hypothesis that Imp-actin binding plays a role in phytoplasma motility in its host plant [16].

Several virulence factors have been identified in ‘*Ca*. P. asteris’. When expressed transiently in tobacco (*Nicotiana benthamiana*) or constitutively in transgenic *Arabidopsis thaliana* lines, the small secreted protein Tengu disturbs auxin-controlled gene expression and, as a result, induces proliferation and dwarfism similar to those in phytoplasma-infected plants [17]. Additionally, in ‘*Ca*. P. asteris’, the virulence effector Sap54 induces indeterminate leaf-like flower development in *Arabidopsis* plants [18], whereas the secreted protein Sap11 down-regulates defence hormone biosynthesis by destabilizing plant development regulators, thereby enhancing insect vector reproduction [19,20]. Homologs of these effector proteins have been detected in the genomes of several other phytoplasmas, but not in the nearly complete genome sequence of the FD phytoplasma [21]. Yet, the genome of the FD phytoplasma, like the genome of ‘*Candidatus* Phytoplasma solani’, encodes variable membrane proteins (Vmps) that are thought to be involved in the interactions of the phytoplasma with its leafhopper vector [22,23]. Indeed, these proteins share limited homology and/or structural features, such as an N-terminal signal sequence followed by a stretch of repeated sequences and a C-terminal transmembrane segment, with various bacterial surface proteins, including adhesins [24-26]. Furthermore, studying the variability of *vmp* genes in strains of FD-related phytoplasmas has revealed that phylogenetic clustering of these genes correlates with the ability to be transmitted by a given leafhopper species of the *Deltocephalinae* family [23,27]. In particular, phytoplasma strains belonging to VmpA clusters II and III are transmitted by the FD phytoplasma vector *Scaphoideus titanus*, whereas those of cluster I are not [23].

To further assess the biological function of Vmps in the interactions of phytoplasmas with the FD-phytoplasma leafhopper vector and to circumvent the lack of phytoplasma mutants, we investigated the possibility of mimicking phytoplasmas by constructing recombinant spiroplasmas that display phytoplasmal surface proteins. Unlike phytoplasmas, the plant pathogenic mollicute *Spiroplasma citri* can be cultured in a cell-free medium [28] and has been made amenable to genetic manipulation [29], making it possible to identify genes involved in interactions with host cells [26,30,31]. In particular, we have recently shown that plasmid vectors derived from natural plasmids of *S. citri* GII3 are suitable for the efficient expression of cloned genes [32]. However, with the exception of the surface lipoprotein spiralin [33,34], the expression of heterologous membrane and/or secreted non-lipoyl-modified proteins has not been achieved.

In the present study, we describe the expression of FD-phytoplasma VmpA in *S. citri*. When fused to the signal peptide of the spiroplasma adhesin *S. citri* adhesion-related protein 3d (ScARP3d), the VmpA pre-protein was processed and translocated to the spiroplasma membrane. We also describe the secretion of functionally active β-lactamase, a model protein that we used to show the potential of the recombinant spiroplasma strategy for delivering phytoplasma effector proteins into hosts.

## Methods

### Bacterial strains, culture conditions and transformation

Phytoplasma strain FD92 was originally transmitted to the broad bean (*Vicia faba* var. aquadulce) using *S. titanus* leafhoppers collected in FD-diseased vineyards in southwest France [35], and continuously maintained in broad beans through serial transmission with the alternative leafhopper vector *Euscelidius variegatus* [36]. *S. citri* strain GII3 was originally isolated from its leafhopper vector *Circulifer haematoceps*, which was captured in Morocco [37]. The low-passage, wild-type strain contains seven plasmids, pSciA and pSci1 to pSci6 [38]. *S. citri* 44 was isolated from a stubborn-diseased sweet orange tree in Iran [39]. In contrast to *S. citri* GII3, *S. citri* 44 has no plasmid [40]. Spiroplasmas were cultivated at 32°C in SP4 medium [41], from which fresh yeast extract was omitted. Electrotransformation of spiroplasmas was conducted as previously described [42] using 1–5 μg of purified plasmid or particular ligation mixtures. Spiroplasmal transformants were first selected by plating on solid SP4 medium containing 2–5 μg/ml tetracycline, and they were further propagated in broth medium containing 5–10 μg/ml tetracycline.

### DNA isolation and plasmid constructions

Spiroplasma plasmid DNA was purified from 8–12 ml cultures (approximately 10^9^ colony-forming units (CFU)/ml) using the Wizard SV minipreps DNA purification kit (Promega, Madison, WI, USA). The *S. citri* plasmids pST2 and pST4 were derived from pSci21NT [32] through deletion of the 977-bp *Cla*I-*Bgl*II fragment. Plasmid pST4 differs from pST2 in that it still contains the *Bgl*II restriction site. To construct pBPTS1, a 200-bp *Eco*RI fragment, consisting of the promoter and ribosome binding site (RBS) of the *S. citri tuf* gene fused to the signal peptide sequence of adhesin ScARP3d, was chemically synthesized (Proteogenix, Schiltigheim, France) and inserted into the *Eco*RI-linearized vector pBS+, from which the *Bam*HI site had been removed by a fill-in reaction. Plasmids pSTP1 and pSTP2 were obtained by inserting the 200-bp *Eco*RI fragment of pBPTS1 into the *Eco*RI site of pST2. To obtain pBPTVA, the *vmpA* gene fragment was obtained by PCR amplification of DNA extracted from FD92-infected broad beans [43] with the primer pair VAF1-VAR2 (Table 1), and the *Bam*HI + *Bgl*II-digested amplicon (1,138 bp) was inserted into the *Bam*HI site of pBPTS1. The VAF1-VAR2 *vmpA* amplicon was ligated to the 3,551-bp *Bam*HI-*Bgl*II fragment of pSD6 [44] to yield pBVA3 and pBVA4, depending on the *vmpA* gene orientation. Plasmid pSTVA1 was obtained by ligating the 1,338-bp *Eco*RI fragment of pBPTVA, which contains the signal peptide-*vmpA* gene fusion downstream of the *tuf* gene promoter, to *Eco*RI-linearized pST2. The intact *vmpA* gene, including its own promoter, was PCR amplified from DNA extracts of FD92-infected broad beans with the primer pair VAF3-VAR2 (Table 1), and the 1,661-bp *Bam*HI + *Bgl*II-digested amplicon was inserted into the *Bgl*II site of pST4 to yield pSTVA3 and pSTVA4, and into the *Bam*HI site of pSTP2 to yield pSTVA5 and pSTVA6, depending on the orientation, sense or antisense, respectively, relative to the *tuf* promoter. The β-lactamase gene (devoid of its own signal peptide sequence) was PCR-amplified from pBS+ using primers BlaF1 and BlaR2 (Table 1). Then, the *Bam*HI + *Bgl*II-digested amplicon was ligated to the *Bam*HI-linearized pSTP2 to yield pSTBl1 (sense) and pSTBl2 (antisense). All constructs were verified by sequencing the relevant plasmid regions (Additional file 1: Figures S1 and Additional file 2: Figure S2).

**Table 1:** **Primers used in this study**

**Primers**	**Sequences**	**Target gene**	**Positions**	**Accession numbers**
VAF1	5′-*ATGAT* ***GGATCC***ATTACAGATTTGAGTGGTGT-3′	vmpA*	765-784	LN680870
VAF3	5′-*ATGAT* ***GGATCC***GTTTAACTAATATAAGTTAAACTCTA-3′	vmpA*	242-267	LN680870
VAR2	5′-*TCATA* ***AGATCT***CAAAATAAATCAATAAAAAACTCAC-3′	vmpA*	1874-1896	LN680870
BlaF1	5′-ATGAT***GGATCC***GCTCACCCAGAAACGCTGGTG-3′	bla**	2918-2938	VB0044
BlaR2	5′-ATGAT***AGATCT***CAAGCAGCAGATTACGCGCAG-3′	bla**	1937-1957	VB0044

*vmpA gene of the FD92 phytoplasma.

**β-lactamase gene from phagemid Bluescribe M13 plus (pBS+).

Bold characters indicate endonuclease restriction sites used for cloning.

### Western immunoblotting and colony blot immunoassay

Spiroplasmal proteins were separated by SDS-PAGE and further analysed by immunoblotting, essentially as described previously [45]. Spiroplasmas were pelleted from 50-ml cultures by centrifugation at 25,000 g for 20 min, washed twice in HEPES-sucrose (HS) buffer (8 mM HEPES [pH 7.4], 280 mM sucrose). *Escherichia coli* cells were pelleted from 2-ml cultures by centrifugation at 18,000 g for 5 min, and washed twice in phosphate-buffered saline (PBS) (137 mM NaCl, 2.7 mM KCl, 10 mM Na2HPO4, 1.76 mM KH2PO4). Protein concentrations were determined using the DC protein assay kit (Bio-Rad, Hercules, CA, USA). Protein preparations were mixed with one volume of 2× Laemmli solubilisation buffer. Proteins samples from infectious *E. variegatus* leafhoppers were prepared by grinding 20 insects in 400 μl of Laemmli buffer for 5–10 min using a Potter homogenizer. Proteins were further solubilised by heating at 80°C for 20 min. The insoluble material was removed by centrifugation, and the supernatant was stored at −20°C until use. After separation by 10% SDS-PAGE, proteins were electro-transferred to a nitrocellulose membrane, and those reacting with anti-VmpA primary antibodies were visualized using a goat anti-rabbit immunoglobulin G-alkaline phosphatase conjugate and NBT-BCIP (Sigmafast™, Sigma-Aldrich, St Louis, MO, USA) as the substrate. For direct colony blotting, a dry nitrocellulose filter (0.45-μm pore size) was laid on top of spiroplasma colonies for 5–10 min. Then, the filter was carefully removed and treated as above for Western blots.

Rabbit polyclonal antibodies raised against recombinant VmpA were produced by Covalab (Villeurbanne, France). Expression and purification of FD phytoplasma VmpA (amino acids 38 to 347) tagged with hexahistidine (His6) was conducted as described previously [26], except that the His6 tag was not removed.

### Triton X-114 partitioning of spiroplasma proteins

Separation of amphiphilic and hydrophobic fractions was performed by the method of Bordier [46], essentially as described previously [47]. Spiroplasma cells from 50-ml cultures were collected by centrifugation at 20,000 g for 30 min, washed three times with HS buffer, and lysed by sonication (10 sec at power level 4) using a Vibracell Sonicator VC500 (Sonics Materials, Newtown, CT, USA). A 10% Triton X-114 solution in Tris-buffered-saline (TBS) (10 mM Tris–HCl [pH 7.4], 150 mM NaCl) was added to a final concentration of 1%, and the mixture was incubated at 4°C for 1 h with gentle rocking. After the insoluble material was removed by centrifugation (20,000 g for 40 min 4°C), the supernatant was further incubated at 37°C for 30 min, and centrifuged at 3,000 g for 3 min. The resulting upper aqueous and lower detergent phases were supplemented with Triton X-114 or TBS, respectively, and washed twice by repeating the phase partitioning step. Finally, proteins from the washed detergent phase were concentrated by methanol precipitation prior to SDS-PAGE analysis.

### Immunogold labelling transmission electron microscopy (TEM)

Spiroplasmas from a late-log phase culture (~10^9^ CFU/ml) were collected by centrifugation at 20,000 g for 20 min and gently re-suspended in 1/10 volume of HS buffer. Fixation was achieved at 0°C for 1 h by adding an equal volume of a mixture of 5% glutaraldehyde and 4% paraformaldehyde in 8 mM HEPES pH 7.4. Formvar carbon-coated nickel grids (200-mesh) were floated on drops of fixed spiroplasmas for 20 min at room temperature. Then, the grids were blotted lightly and placed for 45 min on drops of the rabbit serum (primary antibody) diluted 1:100 in TBS buffer containing 1% bovine serum albumin (BSA). After washing twice in TBS + 0.1% Tween 20 and once in TBS, the grids were placed for 45 min on drops of goat anti-rabbit IgG conjugated to 10 nm gold particles (EM GAR 10; BioCell, Cardiff, NJ, USA) as the secondary antibody. Grids were washed with TBS, then with water. Finally, they were negatively stained with 2% phosphotungstic acid (PTA) pH 7, blotted, air-dried, and examined by TEM at 120 kV on a Tecnai G^2^ Spirit (FEI, Eindhoven, The Netherlands).

### Beta-lactamase assays

Production of biologically active β-lactamase by the transformed spiroplasmas was detected by directly spotting 10–20 μl of spiroplasma culture onto a Cefinase disk (Biomérieux, Marcy l’étoile, France). The presence of β-lactamase activity, i.e., the hydrolysis of the chromogenic substrate nitrocefin, was revealed by a change in disk colour from pale yellow to red within a 5–20 min incubation period at room temperature. To assess the secretion of β-lactamase in the culture medium, 20-μl aliquots of the spiroplasma culture was spotted in the centre of SP4 agar plates and incubated at 32°C for 72 h. After 24, 48 and 72 h incubation periods, 200 μl of an *E. coli* DH10B culture (approximately 10^8^ CFU/ml) was overlaid onto the plate and incubated overnight at 37°C. The *E. coli* cells could only grow on SP4 agar plates containing penicillin G (2 × 10^5^ units/ml) if penicillin was inactivated by the β-lactamase produced by the spiroplasmas. Thus, diffusion of β-lactamase in the SP4 agar medium was evidenced by the ability of *E. coli* cells to grow at increasing distances from spiroplasmal colonies.

## Results and discussion

### Heterologous expression of FD phytoplasma VmpA in *S. citri*

**Figure 1:**
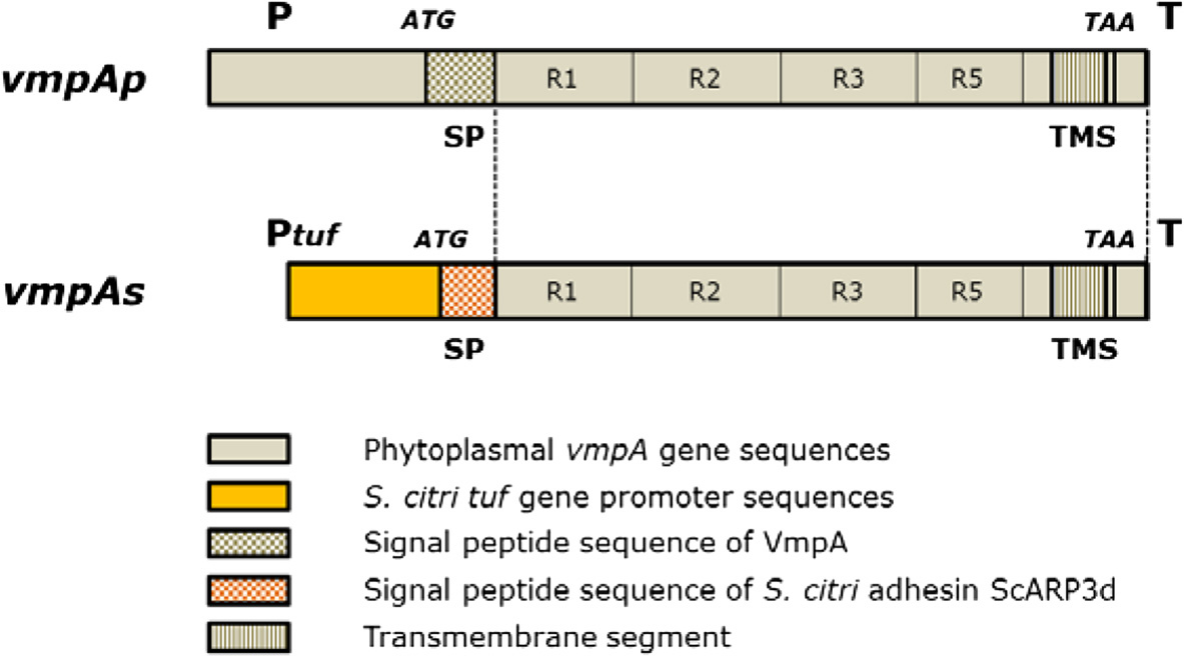
**Schematic representation of the** ***vmpAp*** **and** ***vmpAs*** **gene constructs.** P, *vmpA* gene promoter; P*tuf*, *S. citri tuf* gene promoter; T, transcription terminator; R1 to R5, repeated sequences; SP, signal peptide sequence; TMS, transmembrane segment. ATG start and TAA stop codons are indicated.

To express FD phytoplasma VmpA in *S. citri*, two types of plasmid constructs were engineered and introduced into *S. citri* via transformation. One contained the entire *vmpA* gene, including its own promoter, RBS, and signal peptide sequences (insert *vmpAp*), whereas, in the other, the signal peptide depleted-*vmpA* coding sequence fused to the signal peptide sequence of the adhesin ScARP3d was under the control of the *S. citri tuf* gene promoter and RBS (insert *vmpAs*) (Figure 1 and Additional file 1: Figure S1). Signal peptides are short, hydrophobic peptide sequences at the N-termini of proteins that allow for the translocation of proteins across the membrane via the secretory pathway. The *tuf* gene promoter and RBS sequences were chosen because the *tuf* gene is known to be expressed at a high level in most bacteria [48,49]. In *S. citri*, the presence of −10 and −35 sequences, which are characteristic of bacterial promoters recognized by RNA polymerase associated with its general σ factor, as well as the detection of large amounts of Tuf protein in proteomic analyses, are consistent with the assumption that the *tuf* gene in *S. citri* is constitutively expressed at a high level.

**Figure 2:**
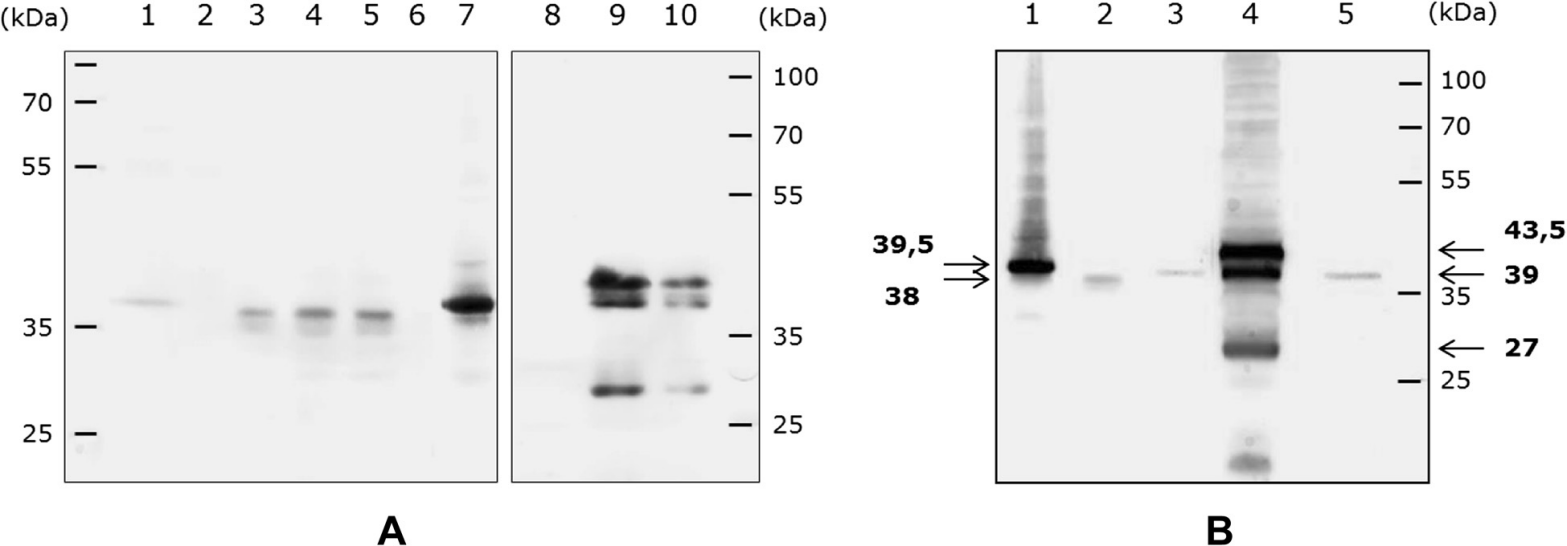
**Western immunoblotting of proteins from FD phytoplasma-infected** ***E. variegatus*** **, and** ***S. citri*** **and** ***E. coli*** **transformants.** The blots were probed with a 1:400 dilution of anti-VmpA rabbit serum. **A** lanes 1, FD phytoplasma-infected *E. variegatus*; 2–7, *S. citri* transformed by pSTP2 (empty vector), pSTVA3–6 [*vmpAp* inserted in sense (pSTVA3 and pSTVA5) and antisense (pSTVA4 and pSTVA6) orientations at two distinct positions (see Additional file 2: Figure S2)], and pSTVA1 (*vmpAs*), respectively; 8–10, *E. coli* transformed by pBPTS1 (empty vector), and pBVA3–4 (*vmpAp*), respectively. Each well was loaded with 100 μg of total proteins in the case of *S. citri* transformants and 20 μg for *E. coli*. **B** lanes 1–2, *S. citri* transformed by pSTVA1 (*vmpAs*) and pSTVA5 (*vmpAp*), respectively; 3 and 5, FD phytoplasma-infected *E. variegatus*; 4, *E. coli* transformed by pSTVA3 (*vmpAp*). Apparent molecular masses of the various VmpA-specific polypeptides are indicated in bold characters.

Depending on the vectors, cloning sites and insert orientation (sense or antisense), various plasmid constructs were obtained (Additional file 2: Figure S2) and introduced into *S. citri* 44 to assess the expression of the *vmpA* gene. In the experiment shown in Figure 2, equal amounts of proteins from spiroplasma transformants carrying pSTVA1, pSTVA3 to 6 or the insert-free vector pSTP2 were separated by 10% SDS-PAGE and probed with anti-VmpA polyclonal antibodies. In all cases, except for pSTVA6 (Figure 2A, lane 6) and the empty vector pSTP2 (Figure 2A, lane 2), a major signal was detected, indicating that the cloned genes, *vmpAp* as well as *vmpAs* were transcribed and translated into polypeptides (Figure 2A, lanes 3–5 and 7). However, a stronger signal was detected in spiroplasmas transformed with *vmpAs* (pSTVA1, lane 7) than those transformed with *vmpAp*, regardless of the *vmpAp* cloning site and gene orientation (pSTVA3 to 5, lanes 3–5), indicating that more VmpA was produced and/or accumulated in these transformants. This finding suggested that, in contrast to the *S. citri tuf* gene promoter, the phytoplasmal *vmpAp* promoter was poorly recognized by the transcription machinery of the spiroplasma. This hypothesis would also account for the failure to detect VmpA in pSTVA6 transformants (Figure 2A, lane 6), in which the *vmpAp* gene was in the antisense orientation to the *S. citri tuf* gene promoter, which is in contrast to the situation in pSTVA5 transformants (see Additional file 2: Figure S2).

Figure 2 also shows that the VmpA-specific polypeptides (VmpAp, Figure 2A, lanes 3–5; and VmpAs, Figure 2A, lane 7) had apparent molecular masses close to that of mature VmpAp (39 kDa) detected in the FD phytoplasma-infected insects (Figure 2A, lane 1). These results suggested that, in both cases, the signal sequences were cleaved (Figure 2A, lanes 3–5 and 7). When expressed in *E. coli*, the *vmpAp* gene yielded three distinct VmpAp polypeptides, the sizes of which corresponded to the pre-protein (43.5 kDa), the predicted mature protein lacking its signal peptide (39 kDa) and an additional cleavage product (27 kDa), respectively (Figure 2A, lanes 9 and 10). A more accurate comparison of the sizes of the various VmpA polypeptides produced in *S. citri*, in *E. coli* and in the FD phytoplasma–infected insects is provided in the Western immunoblot shown in Figure 2B, where the various samples were loaded on the same gel. In *S. citri* transformants, detection of a VmpAp polypeptide of 38 kDa, compared with the 43.5 kDa pre-protein predominantly detected in *E. coli*, supported the hypothesis that the signal sequence of VmpAp was cleaved in spiroplasmas. In *S. citri*, however, the major VmpAp-specific signal had an apparent molecular mass of 38 kDa, slightly lower than the mature VmpAp detected in the insects, suggesting that the VmpAp pre-protein was cleaved in a different way and/or at a different site in spiroplasmas (Figure 2B, lanes 2 and 3). In contrast to VmpAp, the apparent molecular mass (39.5 kDa) of the VmpAs polypeptide detected in the spiroplasmal transformants carrying *vmpAs* (Figure 2B, lane 1) was consistent with the calculated molecular mass (39.5 kDa) of the predicted mature VmpAs, i.e., after cleavage of the spiroplasmal signal peptide. In this case, nearly no pre-protein was detected, indicating that only the mature protein accumulated in the spiroplasmas. This outcome was expected based on previous studies by Berg and co-workers, which showed that the *S. citri* adhesin SARP1 (which is highly similar to ScARP3d) possesses a cleavable signal peptide [50]. As a whole, these results indicate that the secretion machineries and/or signal peptidases of *S. citri* and FD phytoplasma work differently. In agreement with this statement, predictions of FD phytoplasma secreted proteins from the genome sequence revealed that their signal peptides were more than 30 amino acids long, while those of *S. citri* consisted of 23–25 amino acids [21,51]. Signal peptides ranging from 30–34 amino acids are not specific to FD phytoplasma, as they also occur in other phytoplasmas. For example, in ‘*Ca*. P. asteris’, signal peptides of 31, 32 and 33 amino acids have been reported for the well-characterized effectors Sap11, Tengu and Sap54, respectively [17,18,52].

In addition to differences between the signal peptides of secreted proteins, the *S. citri* and FD phytoplasma genomes also contain different sets of secretion-related genes [53]. For example, the FD phytoplasma gene flado_0234_0017, which has a typical S24-S26 peptidase I domain, has no counterpart in *S. citri*. This is consistent with the finding that cleavage of the VmpAp signal peptide occurred at the same position in both FD phytoplasma and *E. coli* (though only partially in this case), but not in *S. citri* (see Figure 2B). Conversely, the protein translocase subunit SecDF and the protein-export membrane protein SecG of *S. citri* have no counterpart in the available sequences of the FD phytoplasma genome. However, despite the fact that none of the four completed phytoplasma genomes (‘*Ca*. P. asteris’ OY-M, ‘*Ca*. P. asteris’ AY-WB ‘*Ca*. P. australiense’ and ‘*Ca*. P. mali’) encode these proteins, the possibility that SecDF and SecG, or proteins with similar functions, could be encoded by the FD phytoplasma genome cannot be fully excluded.

### VmpAs is exposed at the spiroplasma surface

**Figure 3:**
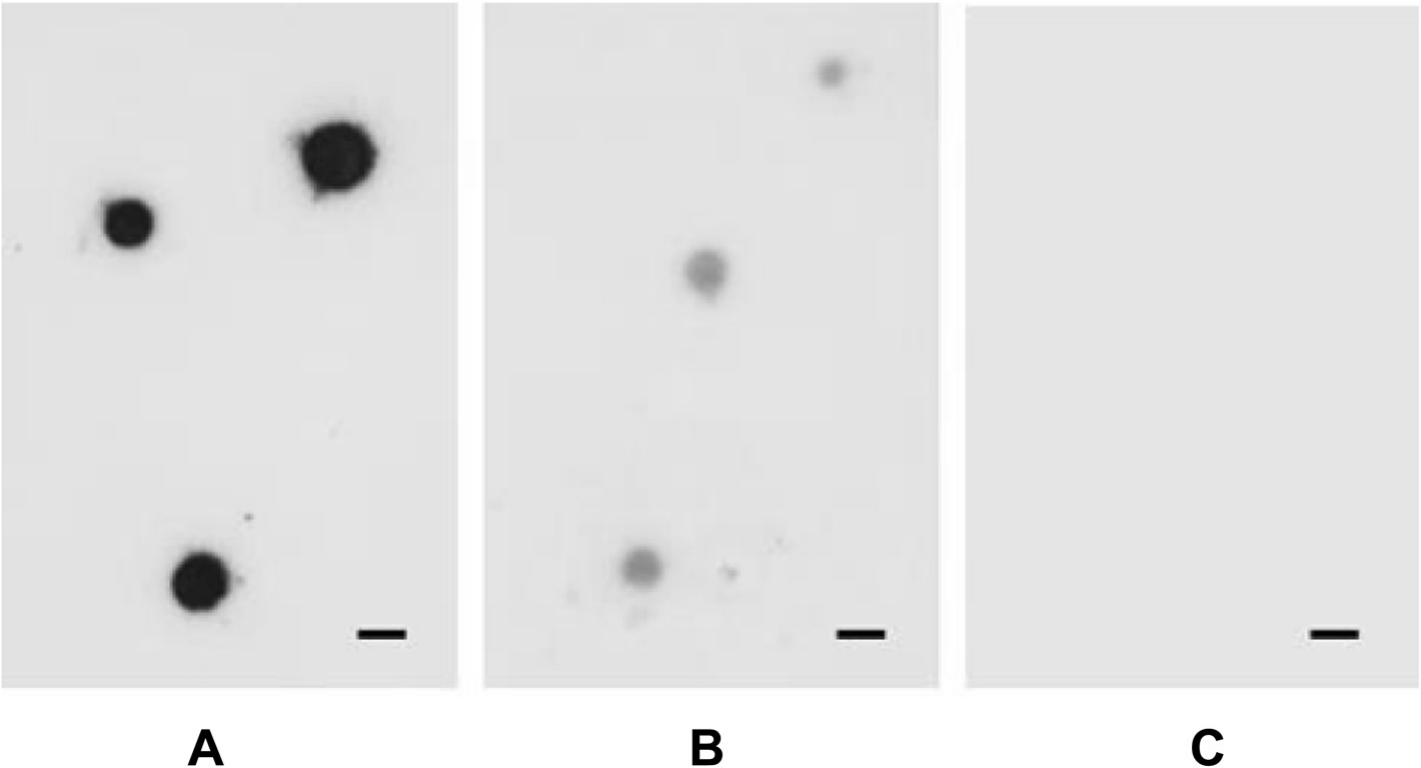
**Direct colony immunoblotting of** ***S. citri*** **transformed by pSTVA1 (** ***vmpAs*** **, A), pSTVA5 (** ***vmpAp*** **, B), and the insert-free vector pSTP2 (C).** The blots were probed with anti-VmpA rabbit serum. Scale bar represents 50 μm.

When analysed by direct colony immunoblotting using anti-VmpA rabbit serum, colonies of the spiroplasmal transformants carrying *vmpAs* strongly reacted with the antibodies, whereas those carrying *vmpAp* were weakly stained, and those carrying the insert-free plasmid were not stained (Figure 3A–C). These results indicated that VmpAs, which has the spiroplasmal signal peptide, was mostly translocated to the spiroplasma cell surface. Once the signal peptide was cleaved, mature VmpAs was probably anchored to the spiroplasma membrane by its C-terminal transmembrane segment, as predicted from the amino acid sequence and as has been reported for *S. citri* adhesins [26,54]. In *S. citri* transformants carrying *vmpAp*, detection of the protein by direct colony immunoblotting suggested that VmpAp also was secreted at the spiroplasma surface. In this case, the weakness of the signal probably resulted from the low level of production of the pre-protein, rather than the failure to be translocated and anchored to the spiroplasma membrane (see Figure 2A). However, the possibility that most of the VmpAp pre-protein produced in the spiroplasma could be rapidly degraded due to the failure to be correctly translocated to the membrane cannot be excluded.

**Figure 4:**
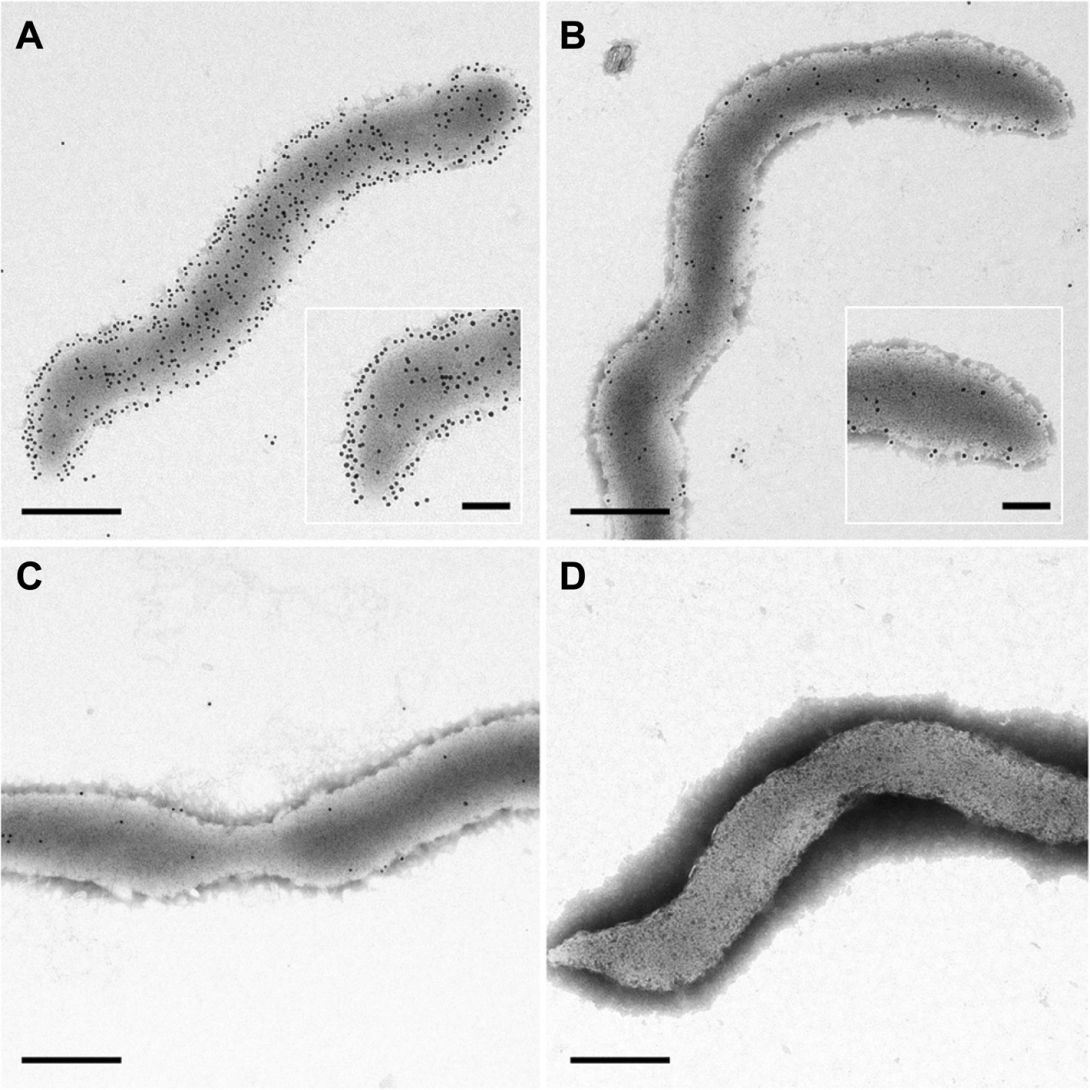
**Immunogold labelling transmission electron microscopy of** ***S. citri*** **transformants.** Labelling of pSTVA1 (*vmpAs*, **A, B** and **D**) and pSTVA5 (*vmpAp*, **C**) transformed spiroplasmas with anti-*S. citri* rabbit serum (**A**, positive control), anti-VmpA rabbit serum **(B and C)**, and in the absence of primary antibodies (**D**, negative control). Scale bars, 250 nm **(A–D)** and 100 nm (insets).

In addition to direct colony immunoblotting, the presence of VmpAs at the spiroplasma cell surface was further illustrated by immunogold labelling and negative staining electron microscopy of spiroplasmal transformants (Figure 4). Despite the absence of cell permeabilisation, many gold particles were detected at the surface of the *vmpAs*-transformed spiroplasmas (Figure 4B), indicating that VmpAs was indeed exposed at the spiroplasma surface. In contrast, very few particles were associated with spiroplasmas transformed by *vmpAp* (Figure 4C), which is in good agreement with the relative amounts of VmpAs and VmpAp detected by Western and colony immunoblotting (see Figures 2 and 3). As expected, spiroplasmas were densely labelled when using an anti-*S. citri* rabbit serum (positive control, Figure 4A), whilst no gold particles were seen in the absence of primary antibody (Figure 4D) and in spiroplasmas transformed with the insert-free vector (data not shown). The images also showed that recombinant VmpAs did not localize at specific sites, but seemed to be randomly distributed along the spiroplasma body, similarly to major *S. citri* surface proteins, such as spiralin and ScARPs, which are involved in the invasion of insect cells [26,31].

### VmpAs is exclusively associated with the spiroplasma membrane

**Figure 5:**
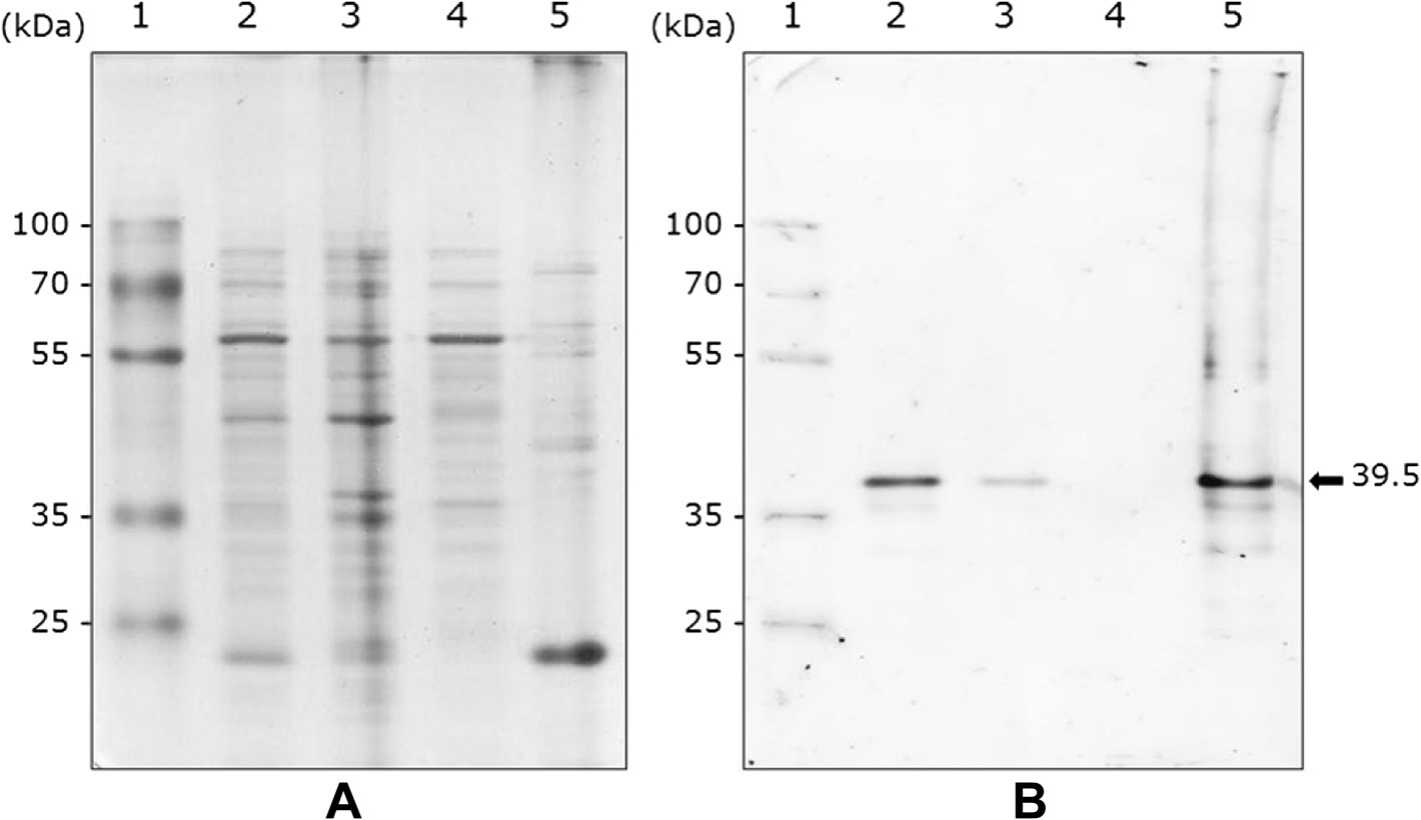
**Triton X-114 phase partitioning of proteins from** ***S. citri*** **transformed by pSTVA1 (** ***vmpAs*** **).** SDS-PAGE **(A)** and western immunoblotting with anti-VmpA rabbit serum **(B)**. Lanes 1–5, molecular mass markers, spiroplasma whole cells, insoluble pellet, aqueous, and detergent phases, respectively. Position and molecular mass of VmpAs are indicated.

To further assess whether VmpAs was mostly anchored to the spiroplasma membrane, total proteins from *vmpAs*-transformed spiroplasmas were submitted to Triton X-114 partitioning, and proteins from both the aqueous and detergent phases were analysed by SDS-PAGE and Western immunoblotting using anti-VmpA rabbit serum as the primary antibody (Figure 5A and B, respectively). As shown in Figure 5A, proteins from the aqueous and detergent phases (lanes 4 and 5, respectively) displayed quite different electrophoretic patterns, indicating an efficient enrichment of the detergent phase in membrane-associated proteins. The immunoblot in Figure 5B shows that most, if not all, of the VmpAs detected in spiroplasma total proteins (Figure 5B, lane 2) partitioned in the detergent phase (Figure 5B, lane 5), as no signal was detected in the aqueous phase (Figure 5B, lane 4). Together with colony and Western immunoblotting, these results further supported that, in the recombinant spiroplasmas, *vmpAs* was transcribed and translated into a polypeptide which was efficiently translocated and anchored to the spiroplasma membrane with concomitant cleavage of the signal peptide. There are many examples describing the heterologous expression of mollicute proteins [26,52,55-60]. In most cases, hydrophilic domains of the proteins were over-expressed in *E. coli* to produce specific antibodies. However, only a few studies have dealt with the heterologous expression of full-length membrane proteins, most of which are lipoproteins that lack a transmembrane domain. For example, heterologous expression of the *S. citri* lipoprotein spiralin has been achieved in *E. coli* [61], *Acholeplasma laidlawii* [62] and *Mycoplasma capricolum* [63]. In these studies, however, complete processing of the pre-protein was only observed in *M. capricolum*, a mycoplasma belonging to the same phylogenetic group as *S. citri* [64,65]. Therefore, the *S. citri* expression system we describe here is the first one in which a transmembrane protein of a non-closely-related mollicute, specifically FD phytoplasma VmpA fused to a spiroplasmal signal peptide, was correctly processed and optimally translocated to the host membrane.

### The signal peptide of *S. citri* adhesin ScARP3d promotes the secretion of free β-lactamase

**Figure 6:**
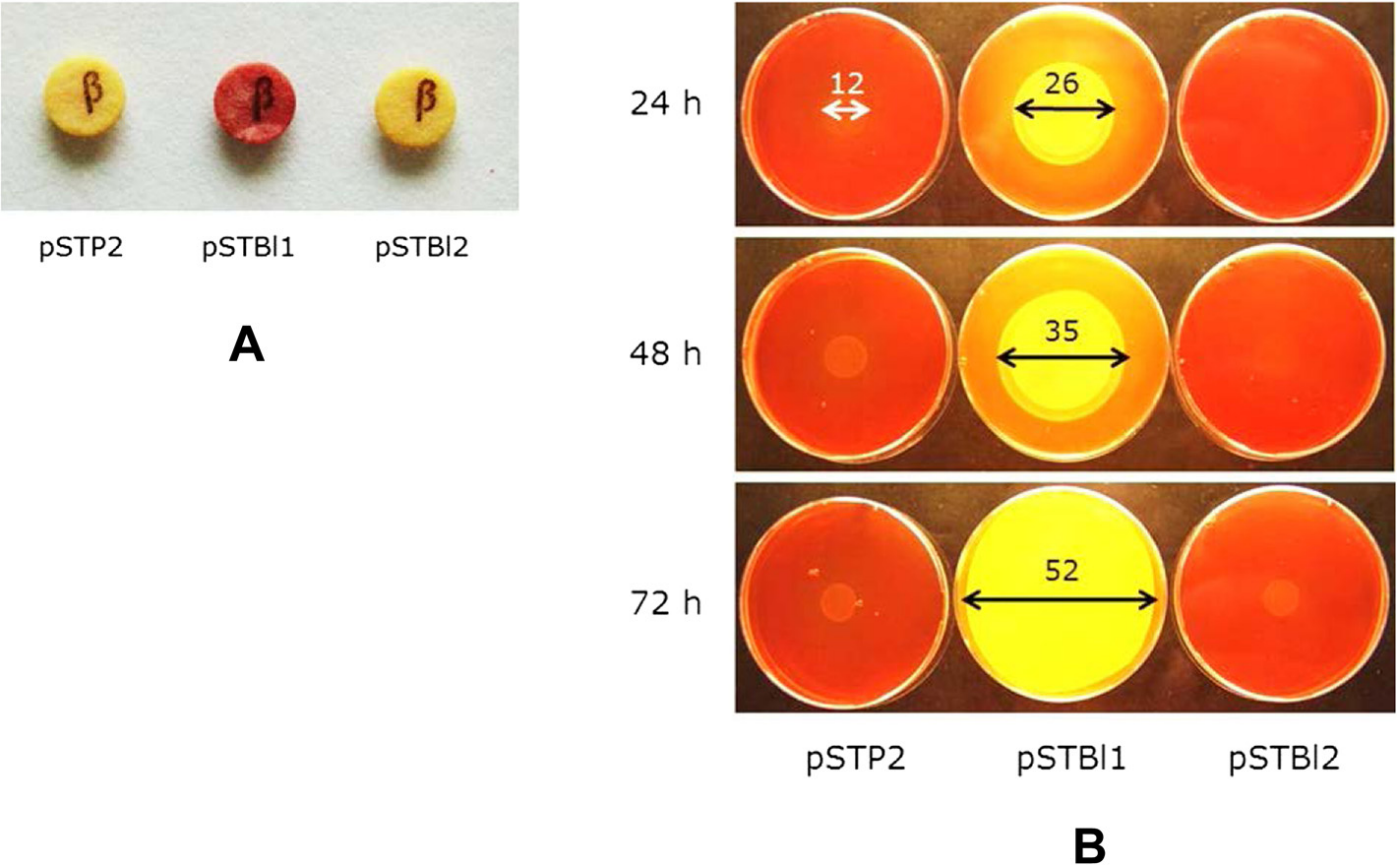
**Production of the** ***E. coli*** **β-lactamase by** ***S. citri*** **transformants. A**, detection of β-lactamase activity by Cephinase™ paper disk assay in spiroplasmas transformed by pSTP2 (empty vector), pSTBl1, and pSTBl2. **B**, secretion and diffusion of free β-lactamase in the culture medium at 24, 48 and 72 h post-inoculation. Numbers above the arrows indicate the diameter (in mm) of the *S. citri* (white arrow) and *E. coli* (black arrows) growing areas.

To determine whether *S. citri* could also be used to deliver diffusible phytoplasma effectors into host cells (insect tissues or plant phloem), we examined the ability of the spiroplasmas to secrete β-lactamase into the culture medium. The *E. coli* β-lactamase gene, in which the signal peptide sequence was replaced by that of the *S. citri* adhesin ScARP3d, was inserted into pSTP2 downstream of the spiroplasma *tuf* gene promoter, and the resulting plasmid, pSTBl1 was introduced into *S. citri* 44 by electro-transformation. The production of β-lactamase by the spiroplasmal transformants was first detected using a Cefinase^TM^ paper disk assay. As indicated by the colour change from pale yellow to red, *S. citri* transformants carrying pSTBl1 produced functional β-lactamase, whereas those transformed by pSTBl2 (which has the insert in the opposite orientation) or the insert-free plasmid pSTP2 did not (Figure 6A). In contrast to VmpAs, β-lactamase has no C-terminal transmembrane segment and, consequently, cleavage of the signal peptide should result in the release of free β-lactamase into the culture medium. To test this hypothesis, recombinant spiroplasmas were spotted in the centre of SP4 agar plates containing Penicillin G and grown for 24, 48 and 72 h before β-lactamase activity, i.e., the inactivation of penicillin, was assayed, as evidenced by the ability of penicillin-sensitive *E. coli* cells to grow on these plates. As shown in Figure 6B, a lawn of contiguous *E. coli* colonies was observed in the case of pSTBl1-transformed spiroplasmas (middle column), but not in those carrying the insert-free plasmid pSTP2 (left column) or pSTBl2 (right column). Notably, the size of the growing area of the *E. coli* cells (Figure 6B, black arrows) was considerably greater than that of the spiroplasma colonies (Figure 6B, white arrow) as soon as 24 h after spiroplasma inoculation, and the entire plate was covered by 72 h. These results clearly indicated that a significant amount of β-lactamase was released into the medium, and that the polypeptide synthesized by the recombinant spiroplasmas was functionally active. As a whole, these experiments strongly suggest that recombinant spiroplasmas are appropriate vehicles for the delivery of diffusible proteins.

Virus vectors and plant transformations have been successful approaches for characterizing phytoplasma effectors. In ‘*Ca*. P. asteris’, the expression of effector candidate genes, such as *sap11* or *tengu* in transgenic *Arabidopsis* lines, resulted in morphological changes reminiscent of phytoplasma infection, and virus-based high level expression of Sap11 enabled the detection of interactions with unstable proteins, such as transcription factors [17,19,66]. Because spiroplasmas and phytoplasmas share the same ecological niches, in particular the phloem sieve elements in which they are introduced by leafhopper vectors, the recombinant spiroplasma strategy might serve as a new approach for characterizing phytoplasma protein effectors, with the advantage that it better reflects phytoplasmal infection.

## Conclusions

Phytoplasma genomes encode several types of secreted proteins, including surface proteins, such as immunodominant membrane proteins (Imp) and variable membrane proteins (Vmp), which are thought to play critical roles in the colonization of the vector insect [14,22,23] and effector proteins, some of which have been shown to alter plant development [17,19,20,67].

In this study, the construction of recombinant spiroplasmas that express the variable membrane protein VmpA of FD phytoplasma at the cell surface has been achieved.

Based on the same strategy, i.e., the combination of the cloned gene fused with the signal peptide sequence of the *S. citri* adhesin, the secretion of free, biologically active β-lactamase has also been demonstrated. At a time when phytoplasmas are still not cultivable, the present study can be seen as a proof of concept of an innovative approach, namely the use of recombinant spiroplasmas that mimic phytoplasmas, for investigating the interactions of phytoplasmas with their hosts. Compared with *in vitro* studies with purified recombinant proteins, such an approach confers the advantage of studying the interactions *in vivo*, with phytoplasmal membrane proteins that have native topologies at the surface of a living bacterium. We have previously reported the role of *S. citri* surface proteins, such as spiralin and adhesins, in the invasion of cells of its leafhopper vector [26,31]. Knowing that *S. citri* has the capability to colonize *E. variegatus* [68], the experimental vector of the FD phytoplasma, studying the roles of diverse phytoplasma Vmps in the adhesion/entry of recombinant spiroplasmas into cells of the FD phytoplasma vector, will provide a better understanding of how Vmps contribute to the specificity of insect transmission.

## Additional files

Additional file 1: Figure S1. A - Nucleotide sequence of the 200-bp *EcoRI* fragment comprising the *tuf* gene promoter and ribosome binding site (RBS) fused to the signal peptide of the *S. citri* adhesin ScARP3d. The −10 and −35 regions, as well as the RBS and ATG start codon, are underlined. The signal peptide sequence is highlighted. The *Bam*HI and *Eco*RI restriction sites are italicized. B - Partial nucleotide sequence of the FD phytoplasma *vmpA* region. The RBS, ATG start and TAA stop codons are underlined, as are the palindromic sequence of the putative transcription terminator. The predicted signal peptide is highlighted. Positions of primers VAF1, VAF3 and VAR2 are indicated. Additional file 2: Figure S2. Partial restriction map and gene organization of plasmids used in this study. Plasmids, the names and sizes of which are indicated, are not drawn to scale. pE, *S. citri* pSci2 gene encoding the replication protein; hp, hypothetical protein gene; soj, partitioning protein gene; tetM, tetracycline resistance gene from Tn*916*; AmpR, ampicillin resistance gene; mob, plasmid mobilization protein; Ps (light grey), *S. citri spiralin* gene promoter; Pt (orange), *S. citri tuf* gene promoter and RBS sequences fused to the signal peptide sequence of the *S. citri* adhesin ScARP3d; Pv (dark grey) promoter of the FD phytoplasma *vmpA* gene; VmpAp, coding sequence of the *vmpA* gene; VmpA*s*, coding sequence of the FD phytoplasma *vmpA* gene devoid of its signal peptide sequence and fused to that of the *S. citri* adhesin ScARP3d; BlaS, coding sequence of the *E. coli* β-lactamase devoid of its signal peptide sequence and fused to that of the *S. citri* adhesin ScARP3d; Bla, β-lactamase coding sequence lacking the signal peptide sequence.

## Abbreviations

FD: Flavescence dorée of grapevine
HS: HEPES-sucrose
RBS: Ribosome binding site
ScARP: *S. citri* adhesion-related protein
PTA: Phosphotungstic acid
SDS-PAGE: Sodium dodecyl sulphate polyacrylamide gel electrophoresis
TBS: Tris-buffered saline
VmpA: Variable membrane protein A
