# Supplementary material for: Heterologous expression and processing of the flavescence dorée phytoplasma variable membrane protein VmpA in Spiroplasma citri
Source: BMC Microbiol. 2015 Apr 2;15:82. doi: 10.1186/s12866-015-0417-5 (PMC4392738; doi:10.1186/s12866-015-0417-5)
Supplement: Additional file 1: Figure S1. — A - Nucleotide sequence of the 200-bp EcoRI fragment comprising the tuf gene promoter and ribosome binding site (RBS) fused to the signal peptide of the S. citri adhesin ScARP3d. The −10 and −35 regions, as well as the RBS and ATG start codon, are underlined. The signal peptide sequence is highlighted. The BamHI and EcoRI restriction sites are italicized. B - Partial nucleotide sequence of the FD phytoplasma vmpA region. The RBS, ATG start and TAA stop codons are underlined, as are the palindromic sequence of the putative transcription terminator. The predicted signal peptide is highlighted. Positions of primers VAF1, VAF3 and VAR2 are indicated. [file 12866_2015_417_MOESM1_ESM.docx]

**Figure S1.**

**A -** Nucleotide sequence of the 200-bp *EcoRI* fragment comprising the *tuf* gene promoter and ribosome binding site (RBS) fused to the signal peptide of the *S. citri* adhesin ScARP3d. The -10 and -35 regions as well as the RBS and ATG start codon are underlined. The signal peptide sequence is highlighted. The *Bam*HI and *Eco*RI restriction sites are italicized.

***AATTC*GTTTAATCTACCATTACTA**

**-35 -10**

**AAAAAAGATTGCAAAGTAAAAAGAATTAATTTACAATAGTTATGACATTTGTC**

**RBS**

**TATTAATTAAAAATATAGGAGGAAAAGATTAAA ATG AAA AAA TTA TTA**

**M K K L L**

**AGT ATC TTA ACT ATA TCA ACA TTA ACA ACG AGC ATA CCC**

**S I L T I S T L T T S I P**

**GCT CCG TTG CTT GCC AAC ACA CCC GCA *GGA TCC* AT*G***

**A P L L A N T P A G S …**

**B -** Partial nucleotide sequence of the FD phytoplasma *vmpA* region. The RBS, ATG start, and TAA stop codons are underlined as well as the palindromic sequence of the putative transcription terminator. The predicted signal peptide is highlighted. Positions of primers VAF1, VAF3, and VAR2 are indicated

**AATCTTTTGCCCCTTATAATACTAATTTGACTTGGTATCAAATTTTTATTAAG**

**ATTGCCAAATTAAGAACAGTTAATATTAAACAATTAGATTTAGCGCTTTATAA**

**TCAACTTTTTCAATGGTATAAAGTTAATTTTATGCGTTTAAACAAACAAGGTT**

**CTTTAAAACCTTATCAACTGGATATGGTTAAAGTTATAAAATATTTTAGTAAA**

**VAF3->**

**TTATTAAATTTTTAATTTTAATTTATAGAGTTTAACTAATATAAGTTAAACTC**

**TATTTTTTTAGAAAATAAATTTTTTTTGATAAAAATTTTATTTTTAAAATAAC**

**TATTTAAAACTTCAAATTAATTTGATTATTTAATACTTTTTTAAACTAAGAAA**

**ATATTTTATGAATTAAAAAAGTATTTAAATTTTGAAAAAATATCAAAAAAGTA**

**TTTTTTTATTGTTATTATTAATTATAAAATGATAAAAAGGATAAAAGACAATT**

**TCATTATTTTAATATAAAAATTTTTTATCATAATATTTTTTAA*GAATTC*ATAA**

**ATTATTCAATTTTTTTTGTTTTTTTTTATTGTAAAATTAATATGTAGAAGTAA**

**TTTTATAGAAATTATTCTCACAATTTTGAAAATATATTTAAAACAATATTTAA**

**RBS**

**TTAGATAGGAAAACAAA ATG ATA GAA AAA CAA ATG AAT AGA AAA**

**M I E K Q M N R K**

**ATT AAT ATT CAA AGT TTT ATA TCT TTA ATT TTT GTT TTT**

**I N I Q S F I S L I F V F**

**ATG TTT TTA TTT TTA AAT GTT TTT TAT TTA ACA CAA ATA**

**M F L F L N V F Y L T Q I**

**VAF1->**

**AAA GCT ATT ACA GAT TTG AGT GGT GTT TTA TTA AAA AAA**

**K A I T D L S G V L L K K**

**GAT TTA GGT GAA ATT ACA TCT AAA GAT TTA AAA GTT ACC**

**D L G E I T S K D L K V T**

**AAA GAA GAA ATT ATA AAT CAA ATT AAA GAA AAA AAT CCA**

**K E E I I N Q I K E K N P**

**:::::::::::::::::::::::::::::::::::::::::::::::::::**

**GAT GAT TTT CAA GGC GAA GTA GAA GTT GAG TTT ACT GTT**

**AAG AAA AAA AGT TTT TTT AGT TTA TTT ACA ATA GCT ATT**

**ATT TTA AGT ATA GGT GTA GCA TCT TTA GCT TTA TTA GGT**

**TAT TAT TTT TAT GAA AAA AAC AAT AAA AAA ATA GAT TAA**

**Transcription terminator**

**AATTTATTTTGAGTATATTTTAGATCGAATAATTAATTTTTTAATTATTCGAT**

**<-VAR2**

**TTTTTTTATATAAATAATTGTGAGTTTTTTATTGATTTATTTTGAAATCAAAA**

**TATAATATTAATTGTGTTTTCAAGAATTAAATAAAATTATCGAAATTTAATTT**

**TTTATATTTAAATTAAAGTTGTTAGTTATAAGAATTTACAAG**
