# Supplementary material for: Heterologous expression and processing of the flavescence dorée phytoplasma variable membrane protein VmpA in Spiroplasma citri
Source: BMC Microbiol. 2015 Apr 2;15:82. doi: 10.1186/s12866-015-0417-5 (PMC4392738; doi:10.1186/s12866-015-0417-5)
Supplement: Additional file 2: Figure S2. — Partial restriction map and gene organization of plasmids used in this study. Plasmids, the names and sizes of which are indicated, are not drawn to scale. pE, S. citri pSci2 gene encoding the replication protein; hp, hypothetical protein gene; soj, partitioning protein gene; tetM, tetracycline resistance gene from Tn916; AmpR, ampicillin resistance gene; mob, plasmid mobilization protein; Ps (light grey), S. citri spiralin gene promoter; Pt (orange), S. citri tuf gene promoter and RBS sequences fused to the signal peptide sequence of the S. citri adhesin ScARP3d; Pv (dark grey) promoter of the FD phytoplasma vmpA gene; VmpAp, coding sequence of the vmpA gene; VmpAs, coding sequence of the FD phytoplasma vmpA gene devoid of its signal peptide sequence and fused to that of the S. citri adhesin ScARP3d; BlaS, coding sequence of the E. coli β-lactamase devoid of its signal peptide sequence and fused to that of the S. citri adhesin ScARP3d; Bla, β-lactamase coding sequence lacking the signal peptide sequence. [file 12866_2015_417_MOESM2_ESM.pptx]

## Slide 1
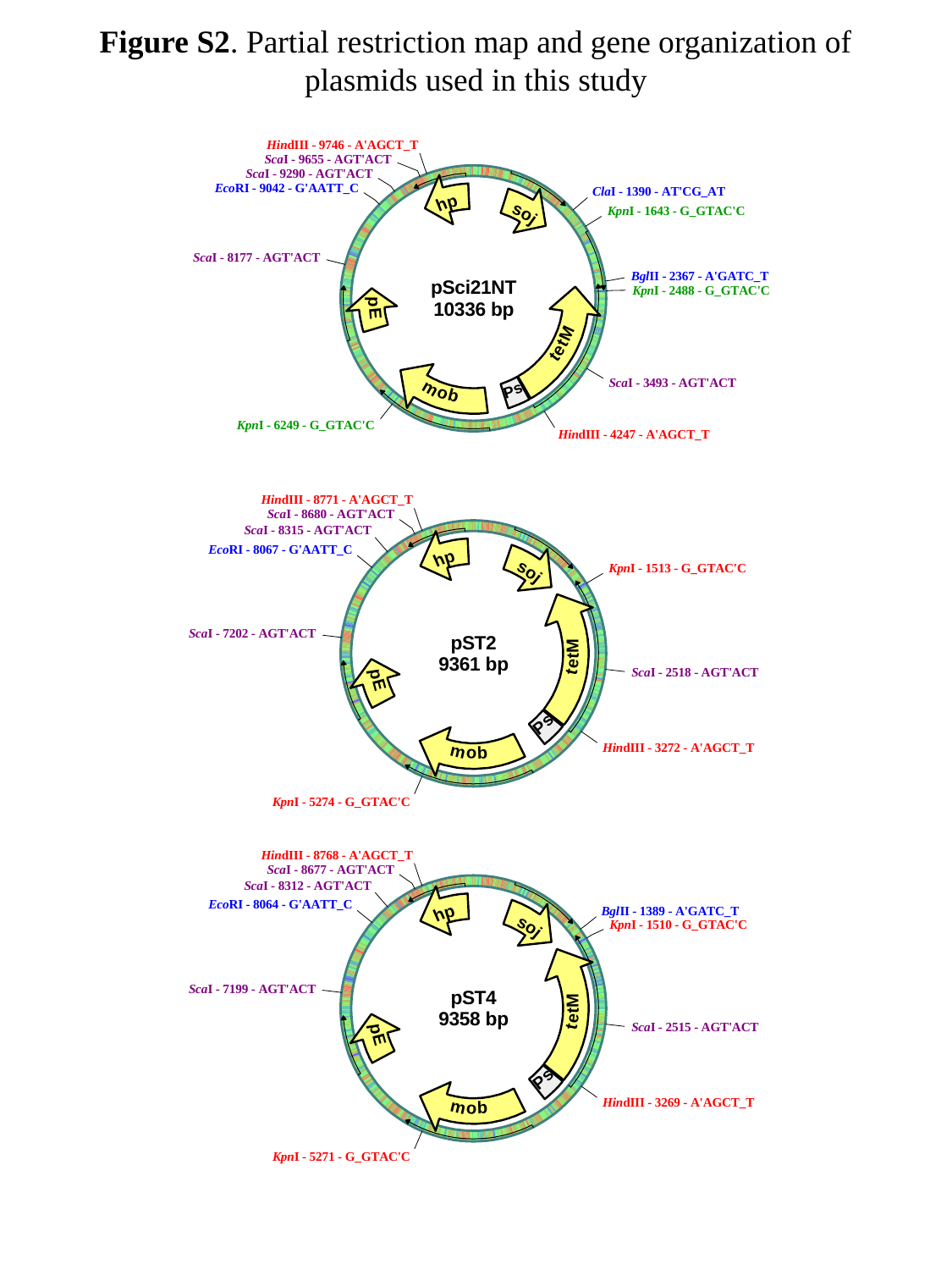

Figure S2. Partial restriction map and gene organization of plasmids used in this study

## Slide 2
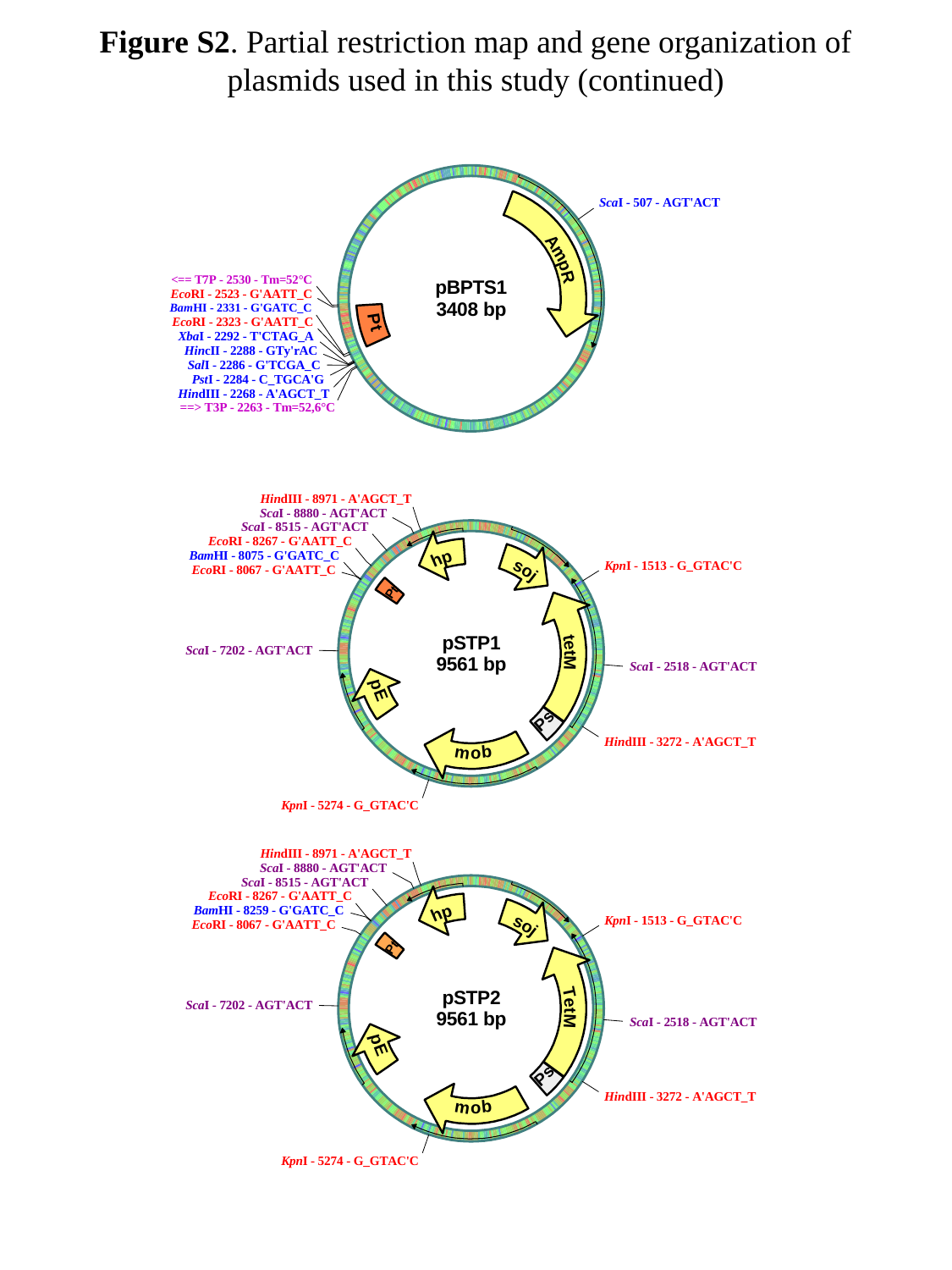

Figure S2. Partial restriction map and gene organization of plasmids used in this study (continued)

## Slide 3
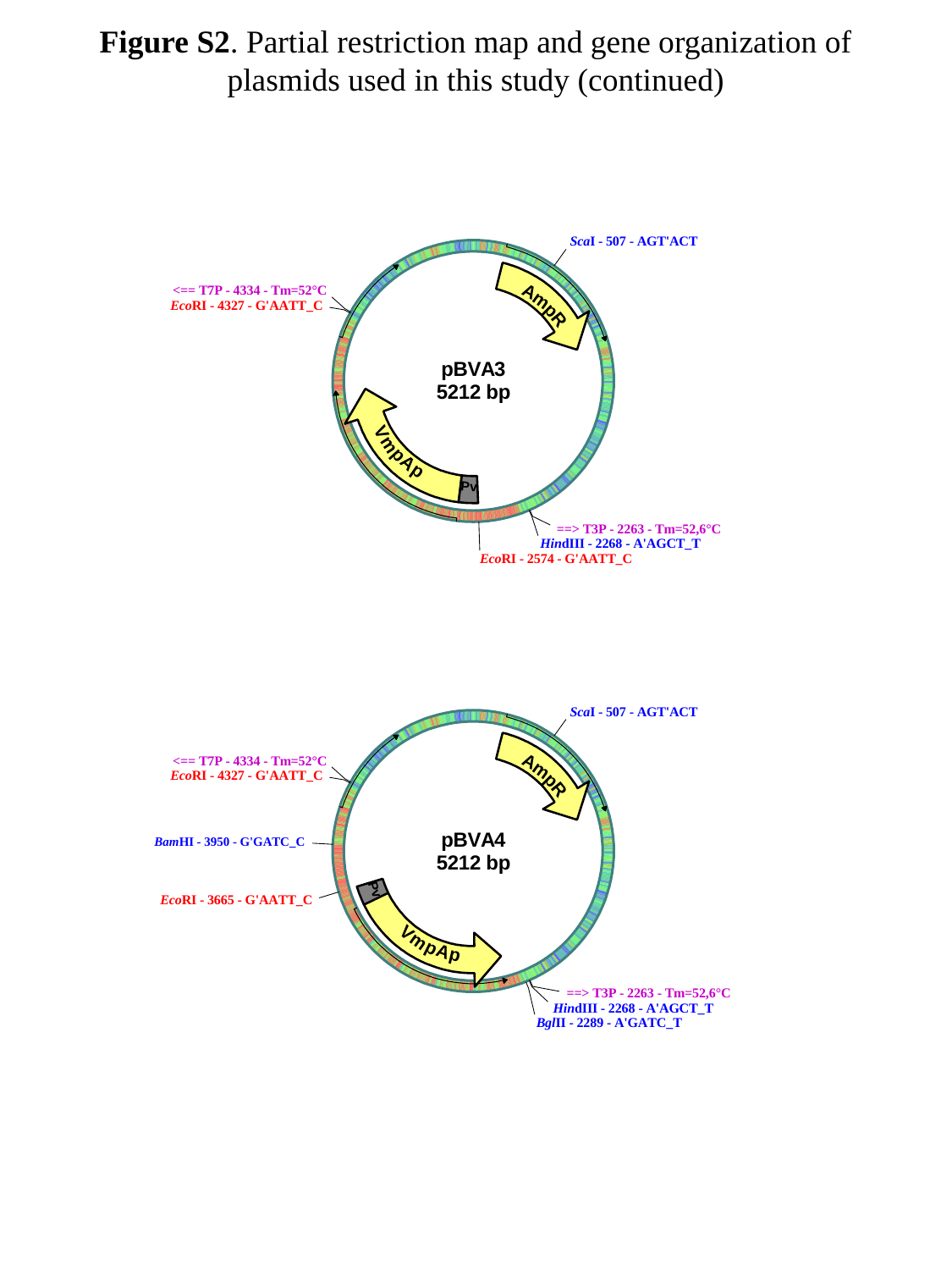

Figure S2. Partial restriction map and gene organization of plasmids used in this study (continued)

## Slide 4
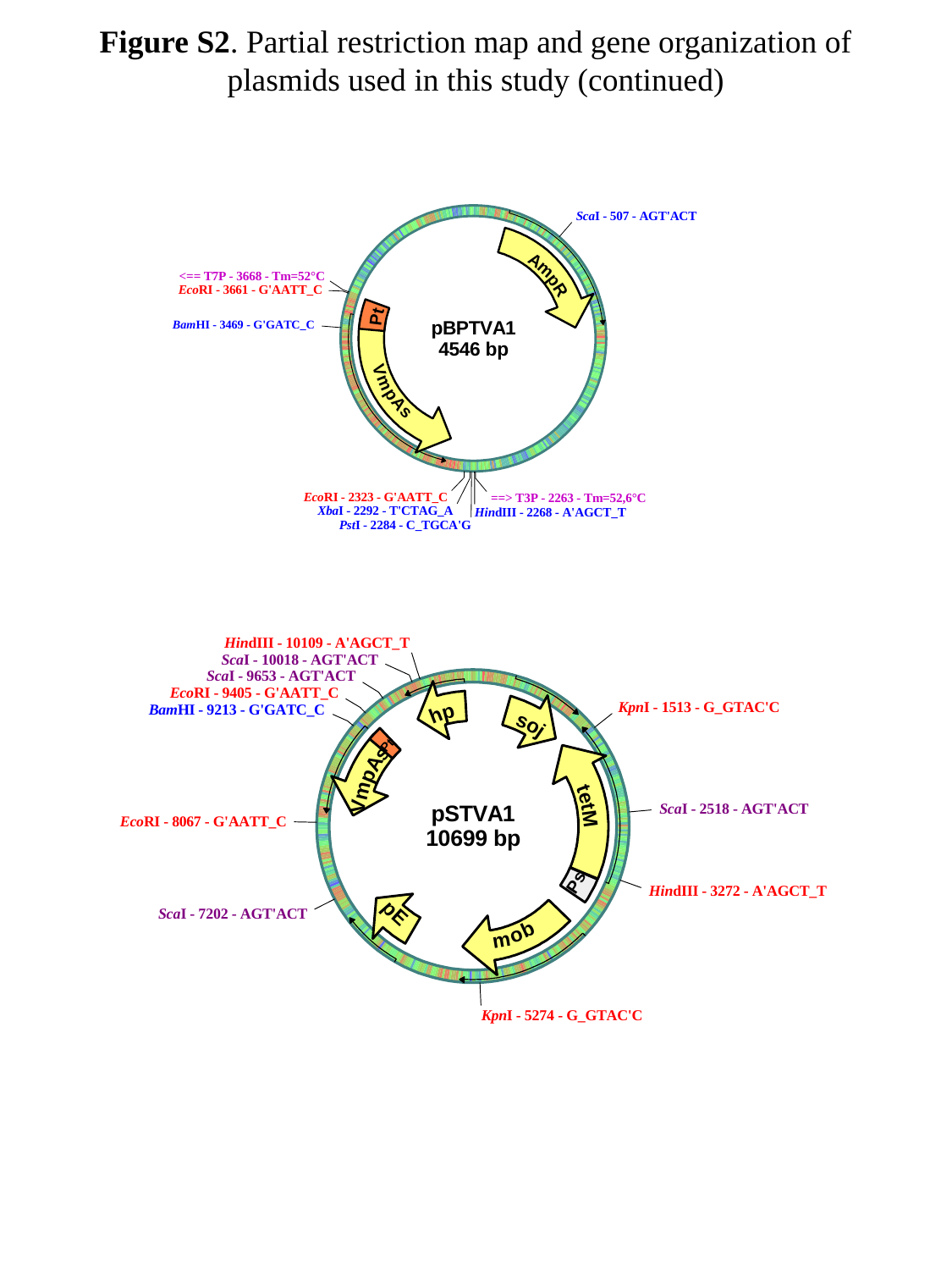

Figure S2. Partial restriction map and gene organization of plasmids used in this study (continued)

## Slide 5
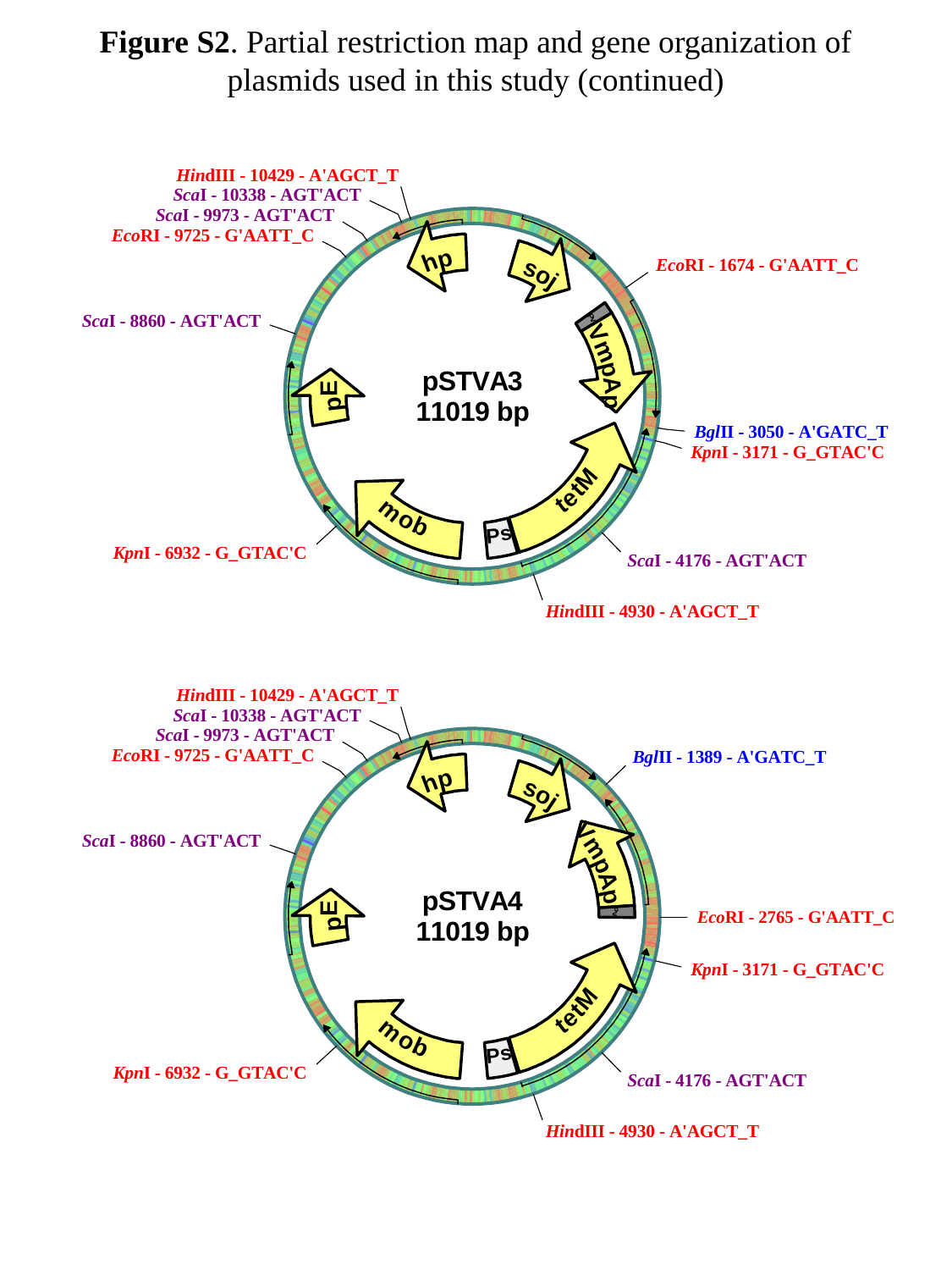

Figure S2. Partial restriction map and gene organization of plasmids used in this study (continued)

## Slide 6
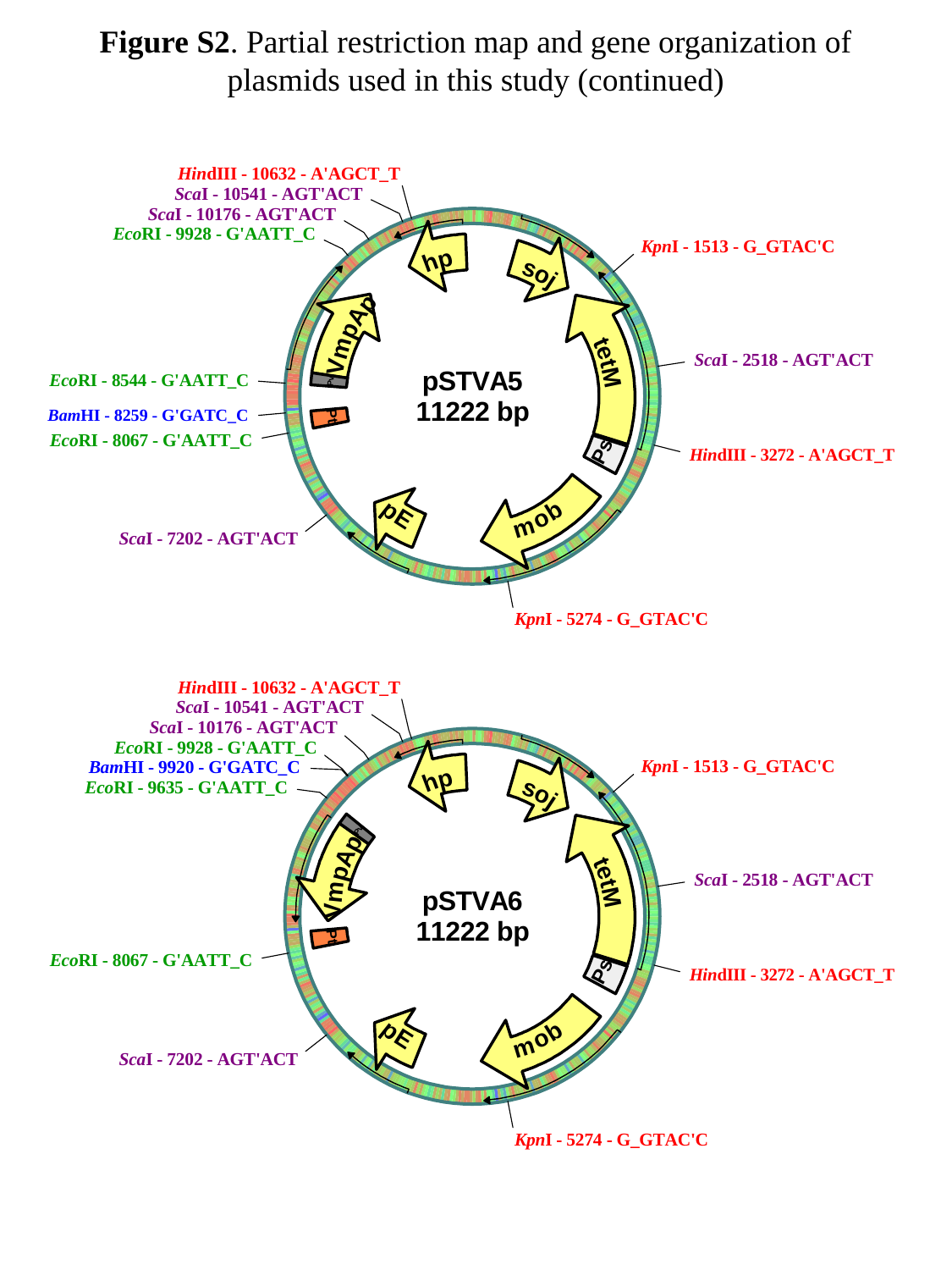

Figure S2. Partial restriction map and gene organization of plasmids used in this study (continued)

## Slide 7
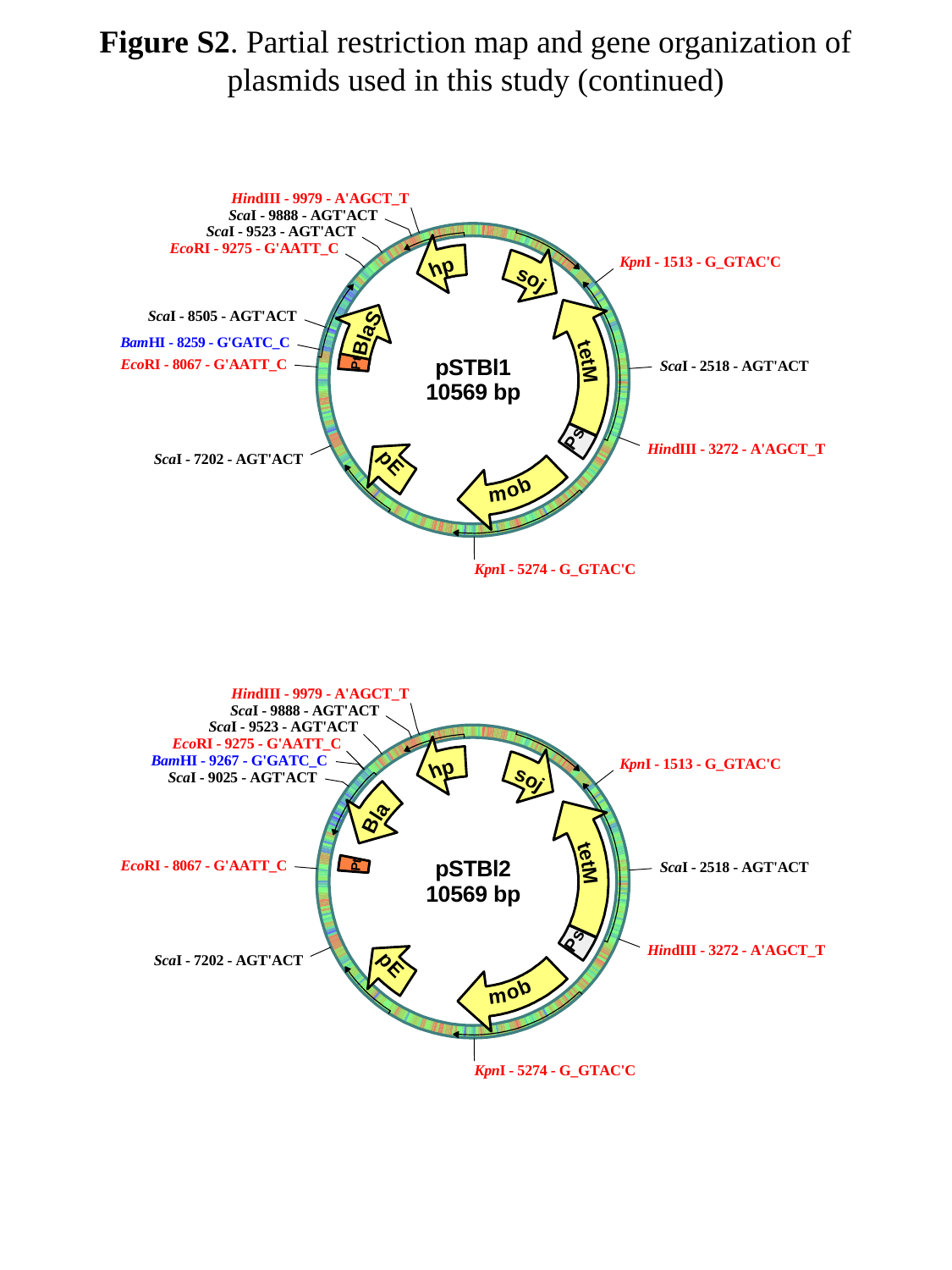

Figure S2. Partial restriction map and gene organization of plasmids used in this study (continued)
